# People living with HIV and COVID-19: A report on 2 clinical cases from South Africa

**DOI:** 10.7196/AJTCCM.2020.v26i2.078

**Published:** 2020-06-15

**Authors:** M Mitha, A P Maharaj, K Nyamande

**Affiliations:** Inkosi Albert Luthuli Central Hospital, Durban, South Africa

**Keywords:** HIV, COVID 19, ART

## Abstract

The impact of HIV in severe acute respiratory syndrome coronavirus 2 (SARS-CoV-2) infection has not been well established. It is uncertain if outcomes are better or worse in these patients compared with COVID-19 patients with diabetes mellitus, hypertension and other chronic diseases. The course and outcome is also unknown in HIV-positive patients who are virally suppressed on antiretroviral treatment (ART) compared with those who are treatment-naive. We present two HIV-positive cases with COVID-19 pneumonia – one virally suppressed and the other newly diagnosed. Both patients had favourable outcomes.

## Case 1

A 31-year-old female initially presented with a 1-week history of flu-like symptoms and had a positive COVID-19 PCR test result on 10 April 2020. She was transferred from the base hospital to the Inkosi Albert Luthuli Central Hospital (IALCH) intensive care unit (ICU) with acute onset respiratory distress. She had a respiratory rate of 30 breaths per minute, blood pressure of 140/75 mmHg and a pulse rate of 107 beats per minute. Her oxygen saturation was 88% on ambient air, increasing to 93% on supplemental oxygen via nasal prongs. Her arterial blood gas (ABG) revealed a pH of 7.51, pCO_2_ of 4.8kPa, pO_2_ of 8.3 kPa and sO_2_ of 90%.

**Fig. 1:**
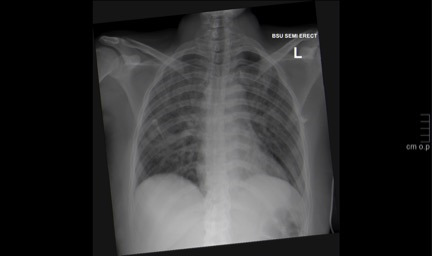
Chest X-ray showing bilateral shadowing in case 1.

**Fig. 2:**
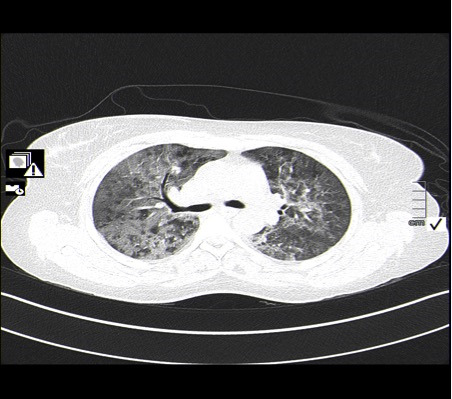
Computed tomography chest scan showing bilateral ground-glass opacities in case 1.

Her chest radiograph demonstrated diffuse bilateral reticular nodular infiltrates and computed tomogography (CT) scans showed diffuse bilateral ground-glass opacities (Figs1 and 2). She was treated with 1 g meropenem 8-hourly, 60 mg subcutaneous enoxaparin twice daily, 50 mg hydrocortisone 6-hourly, as well as zinc supplementation.

She was counseled and tested positive for HIV, with a CD4^+^ count of 3 cells/uL and a viral load of 53 781 copies/mL. She did not require intubation and maintained satisfactory oxygenation on nasal prongs. Antiretroviral therapy (ART) was commenced as well as *Pneumocystis * prophylaxis. She was transferred to the general ward after 3 days. Four days later she did not require supplementary oxygen and was subsequently discharged after a negative COVID-19 PCR test result.

## Case 2

**Fig. 3:**
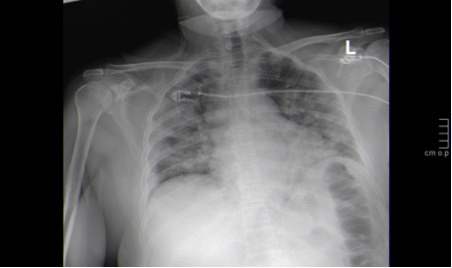
Chest X-ray of case 2. Chest X-ray showing diffuse shadowing bilaterally in case 2.

**Fig. 4:**
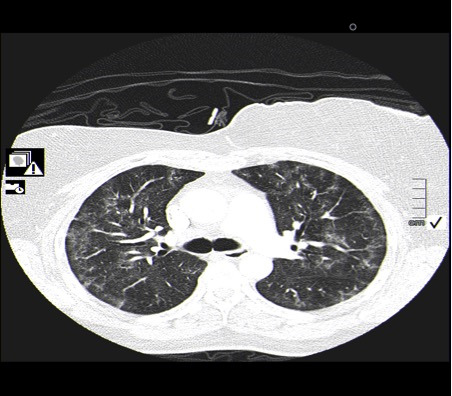
Computed tomography chest scan showing bilateral ground-glass opacities in case 2.

A 55-year-old HIV-positive female on combination ARV therapy for 10 years (tenofovir, emtricitabine and efavirenz), as well as hypertension for 10 years on hydrochlorothiazide, presented acutely with a 1-day history of breathlessness after testing positive 1 week prior for COVID-19. She had a CD4^+^ cell count of 671 cells/uL and undetectable viral load. She was referred from the base hospital to IALCH ICU in severe respiratory distress with a respiratory rate of 35 breaths per minute, blood pressure 133/92 mmHg and pulse rate of 74 beats per minute. Her oxygen saturation on room air was 77% with the following ABG values: pH 7.5; pO_2_ 7.1 kPa; and pCO_2_ 5.9 kPa. Her chest radiograph revealed extensive nodular shadowing bilaterally and CT showed scattered ground-glass opacities bilaterally (Figs3 and 4).

She was placed on a 100% non-rebreather mask and oxygen saturation improved to 95%. She was commenced on 50 mg hydrocortisone 6-hourly, 60 mg enoxaparin twice daily, 1.2 g amoxicillin-clavulanic acid 8-hourly, 240 mg intravenous gentamicin daily, as well as her ART and anti-hypertensive treatment. The patient improved, requiring nasal prongs 2 days later, and a further 3 days later she did not require any supplemental oxygen.

## Discussion

The prevalence of HIV in South Africa is estimated to be 13.9% of total population, which is ~8 million people.^[1]^ The outbreak of COVID-19 is a challenging time for clinicians globally, with new research findings being published daily. There are still many unknown factors regarding the disease and its outcomes in areas with high burdens of HIV. There have been few case series reported in Spain and China where HIV was not identified as an increased risk factor for mortality.^[2,3]^ However, the findings were recorded in countries with a low HIV prevalence and where most patients are on ART.

A recent report published in *Lancet AIDS* documented 5 HIV-positive patients in Spain, 4 of whom recovered and 1 remained in ICU at the time of publication. There were no deaths. The patients were given hydroxychloroquine, lopinavir/ritonavir and other immunomudulatory medications.^[2]^

A study^[3]^ conducted in Wuhan, China, among 1 184 HIV-positive patients revealed that only 6 patients developed COVID-19 and all were on ART. None of these patients required hospitalisation and they recovered fully. It was postulated that it could have been due to impaired immunity leading to a decreased inflammatory response, which also supports the early use of corticosteroids. Both our patients were commenced on hydrocortisone early and both had a good outcome. The authors also postulated that lopinavir/ritonavir might have a protective effect.^[3]^ However, in our patient on ART, lopinavir/ritonavir was not part of the regimen.

A case report^[4]^ from China described an HIV-positive patient with a CD4^+^ count of 32 cells/uL who had a protracted course of disease but still recovered. The patient had also been given a course of steroids.Another case report^[5]^ from Wuhan also had a diabetic patient who was diagnosed with HIV while being admitted for COVID-19. This patient was given lopinavir/ritonavir as well as corticosteroids and made a remarkable recovery. The CD4^+^ cell percentage count was low in this patient.

A case series from Germany described 33 patients, all of whom were HIV-positive on ART with a median CD4^+^ cell count of 672 cells/uL. Only 3 of the patients demised, with the majority recovering. There was no increased mortality and morbidity in their case series.^[6]^

Our cases highlight several unique aspects. Firstly, we had a patient with a very low CD4^+^ count who made a dramatic recovery with supplemental oxygen, steroids and anticoagulants. Then we had a virologically suppressed patient on ART which did not include lopinavir/ ritonavir combination. The patient was given supplemental oxygen, steroids as well as anticoagulation and recovered. This outcome supports the findings by Guo *et al*.^[3]^ and the postulation by Haerter *et al*.^[6]^ that HIV-positive patients have a good outcome owing to their impaired immune responses. Wang *et al*.^[4]^ also suggest that early steroid use is beneficial, which our case reports supports. In our country with a high HIV prevalence, it is promising to note that patients with HIV, whether on ART or not, may not experience increased mortality. Data with large numbers of patients are required to come to a firm conclusion.
